# Long-term treatment of hairy cell leukemia with interferon-α: still a viable therapeutic option

**DOI:** 10.1007/s12254-016-0269-1

**Published:** 2016-06-17

**Authors:** Jan-Paul Bohn, Guenther Gastl, Michael Steurer

**Affiliations:** Department of Internal Medicine V, Medical University of Innsbruck, Anichstrasse 35, 6020 Innsbruck, Austria

**Keywords:** hairy cell leukemia, infectious complications, interferon-α, long-term treatment, relapse

## Abstract

Classic hairy cell leukemia (HCL) is a rare indolent B‑cell-lymphoproliferative disorder, first described as a distinct disease entity in 1958. After more than two decades without effective chemotherapeutic options and a dismal prognosis of less than 5 years, only the introduction of interferon‑α (IFN‑α) allowed for response rates between 80–90 % and survival improvement. Nowadays, however, patients are rarely treated with IFN-α as purine analogues were found to be highly effective in HCL facilitating a near normal life span in most cases. Moreover, novel therapeutic tools for patients with relapsed or refractory disease after purine analogues have emerged such as rituximab and, more recently, vemurafenib. In the absence of long-term safety data for these novel agents, however, IFN-α may still represent a viable therapeutic option when the profound immunosuppressive side effects of purine analogues are to be avoided. We herein report a HCL patient, who has received multiple lines of therapy, including pentostatin, cladribine, and a total of 164 months of treatment with IFN‑α yielding long-term disease control. Our case illustrates that long-term administration of IFN-α with adequate dose-adjustments according to toxicity and disease activity is feasible in HCL and may still be a viable therapeutic option when purine analogues are considered unsuitable.

## Introduction

Classic hairy cell leukemia (HCL) is a rare indolent B‑cell lymphoproliferative disorder. First described as a distinct disease entity by Bouroncle et al. in 1958, HCL inherited a dismal survival prognosis of less than 5 years [1]. In the absence of effective systemic therapeutic options splenectomy was treatment of choice for decades, only allowing for short-lived remissions [2]. The first substantial therapeutic intervention in this previously “untreatable” chronic leukemia was made with the introduction of interferon‑α (IFN‑α) in 1984 with response rates of 80–90 % (≤90 % partial remissions, PR; ≤40 % complete remissions, CR) and extension in survival [3–6]. Only shortly thereafter, however, HCL was found to be highly sensitive for purine analogues, an observation that rapidly altered the natural history of HCL. Facilitating a near normal life span in the majority of patients, purine analogues have become first line therapy for HCL [7, 8]. Inferior in terms of quality and duration of response, nowadays IFN-α is rarely treatment of choice. Moreover, novel therapeutic tools for patients with relapsed or refractory disease after purine analogues have emerged such as rituximab and, more recently, vemurafenib [9–13]. In the absence of long-term safety data for these novel agents, however, induction and maintenance therapy with IFN-α may still represent a viable therapeutic option when the profound immunosuppressive side effects of purine analogues are to be avoided, e. g., in the presence of significant comorbid conditions and ongoing infectious complications. We herein report a HCL patient, who has received multiple lines of therapy, including pentostatin, cladribine, and a total of 164 months of treatment with IFN-α yielding long-term disease control (Table 1).

**Table 1:** Clinical outcome of consecutive treatments in our HCL patient

Line of treatment	Regimen	Dosage	Date	Treatment duration	Response	Progression-free survival	Grade III/IV toxicity
1	Splenectomy	–	05/1982	–	PR	12 months	None
2	Interferon-α	1.5 × 10^6^ U/week	06/1983–06/1986	36 months	PR	36 months	None
3	Pentostatin	4 mg/m²	07/1986–03/1987	8 months^a^	CR	163 months	Recurrent infectious complications due to prolonged neutropenia
4	Interferon-α	4.5–9 × 10^6^ U/week	01/2000–11/2007	95 months	PR	95 months	None
5	Cladribine	0.14 mg/kg/day	11/2007	5 days (1 cycle)	CR	49 months	Recurrent infectious complications due to prolonged neutropenia
6	Interferon-α	4.5 × 10^6^ U/week	12/2011–08/2014	33 months	PR	33 months	None

*CR* complete remission. A complete remission was defined as the morphological absence of hairy cells in blood and bone marrow in combination with complete resolution of cytopenia and organomegaly

*PR* partial remission. A partial remission was defined as a complete resolution of cytopenia in combination with at least 50 % decrease in organomegaly and hairy cell infiltration of the bone marrow

^a^4 mg/m² per week x3, then every other week x3, then once monthly x6

## Case report

The now 80-year-old female patient was diagnosed with HCL at the age of 47 years and treated with elective splenectomy. One year later, she required systemic therapy and received IFN-α induction treatment for 3 months resulting in a PR with residual bone marrow infiltration, which was maintained over 3 years (1.5 × 10^6^ U/week). At the age of 51, the patient was switched to pentostatin resulting in a CR and a progression-free survival (PFS) of 13 years. Due to recurrent serious infectious complications after treatment with pentostatin (febrile neutropenia, urinary tract infections, pneumonias) she was considered unsuitable for further purine analogue treatment. Hence, she again received IFN-α for the next 95 months at varying dosages adjusted according to toxicity and disease activity (4.5–9 × 10^6^ U/week) resulting in a CR. Bone marrow biopsy showed only minimal infiltration (<0.5 %) by hairy cells after 2 years on IFN-α. Nevertheless, IFN-α was stopped due to persisting side-effects, mostly flu-like symptoms and fatigue. Therefore, she received cladribine resulting in a CR of 49 months, but also in prolonged pancytopenia complicated by recurrent infections (febrile neutropenia, urinary tract infections). Thus, when the next line of treatment was necessary, the now 76-year-old patient preferred IFN-α (4.5 × 10^6^ U/week) over purine analogues, resulting in a sustained disease control with fully compensated blood counts but persistent hairy cells in peripheral blood for 33 months. Altogether, our patient has received IFN-α treatment cumulatively for almost 14 years. Long-term disease control was achievable, even after pretreatment with purine analogues. Besides recurrent flu-like symptoms and fatigue, no severe adverse advents such as autoimmune phenomena and no secondary malignancies were observed.

## Discussion

Purine analogues are highly effective in HCL and more convenient to patients than IFN-α considering the single cycle of therapy needed to achieve a durable CR in most cases [7]. However, treatment with purine analogues is often accompanied by severe myelotoxicity, which cumulates with each further cycle of therapy necessary at relapse possibly leading to significant infectious complications. The use of IFN-α in HCL is not commonly associated with severe adverse events, but the CR rates achievable are low and relapse seems inevitable after cessation of the drug [8]. Nevertheless, two small retrospective studies suggest that induction therapy with IFN-α followed by maintenance until disease progression may be of more durable efficacy and result in longer PFS. Long-term tolerability was achievable with only a minority of patients experiencing dose-limiting side effects, which prevented further drug administration [14, 15]. Notably, in previously untreated HCL patients even minimal doses of IFN-α have been shown to be clinically effective and almost free of toxicity [16].

These findings also gain more timeliness in view of novel treatment options provided for HCL patients. As HCL strongly expresses CD20 [17], rituximab has demonstrated efficacy with response rates of 25–80 % in relapsed or refractory patients [9–12]. As IFN-α, however, rituximab is inferior to purine analogues with regard to quality and duration of response and there is no data about maintenance therapy. Moreover, anti-CD20 antibodies may also cause further immunosuppression as well as infusion related reactions [18].

More recently, the usage of vemurafenib demonstrated efficacy in 50 relapsed or refractory HCL patients with confirmed BRAF V600E mutation status [13]. In the absence of clinically significant myelotoxicity, vemurafenib was suggested an adequate therapeutic option in patients with cumulative myelotoxicity due to previous chemotherapies. However, disease progression is frequently observed after cessation of the drug as residual leukemic cells may persist under vemurafenib treatment. Moreover, 14 % of patients experienced secondary skin tumors despite the limitation of vemurafenib administration to 16–18 weeks. Notably, even low-dose vemurafenib may increase the risk of secondary cancers when given continuously [19].

Our HCL patient with recurrent severe infectious complications after purine analogues eventually achieved long-term disease control with IFN-α at varying dosages adjusted to toxicity and disease activity for a total of 164 months. Treatment interruptions resulted in rapid relapses, but the patient remained drug-sensitive despite three courses of IFN-α with total treatment duration of nearly 14 years. With a follow-up of 33 years, no cumulative toxicity and in particular no secondary malignancies— a finding in HCL patients reported to be related to (repeated) purine analogue treatments—have been observed [20].

Despite the novel tools emerged in the treatment of HCL, our case illustrates that long-term administration of IFN-α with adequate dose-adjustments according to toxicity and disease activity is feasible and may still be a viable therapeutic option when purine analogues are considered unsuitable.
